# Non-lactational Infectious Mastitis in the Americas: A Systematic Review

**DOI:** 10.3389/fmed.2021.672513

**Published:** 2021-08-02

**Authors:** Victor Costa Morais Oliveira, Nadia Cubas-Vega, Paola López Del-Tejo, Djane C. Baía-da-Silva, Michel Araújo Tavares, Izabella Picinin Safe, Marcelo Cordeiro-Santos, Marcus Vinícius Guimarães Lacerda, Fernando Val

**Affiliations:** ^1^Fundação de Medicina Tropical Dr. Heitor Vieira Dourado, Manaus, Brazil; ^2^Fundação Hospital Adriano Jorge, Manaus, Brazil; ^3^Programa de Pós-graduação em Medicina Tropical, Universidade Do Estado Do Amazonas, Manaus, Brazil; ^4^Faculdade de Medicina, Universidade Federal Do Amazonas, Manaus, Brazil; ^5^Departamento de Ensino e Pesquisa, Universidade Nilton Lins, Manaus, Brazil; ^6^Rede de Pesquisa em Malária, Instituto Leônidas and Maria Deane, Fiocruz-Amazonas, Manaus, Brazil

**Keywords:** non-lactational mastitis, clinical mastitis, granulomatous mastitis, *Mycobacterium tuberculosis*, epidemiology

## Abstract

**Background:** Non-lactational infectious mastitis (NLIM) is an inflammatory breast disease with broad clinical presentation. Inadequate treatment can lead to chronic infections that cause breast deformities. NLIM information is limited, especially in the Americas. A systematic review and meta-analysis have been conducted here.

**Methods:** Literature search was conducted in three databases (Lilacs, PubMed, and Scielo) on NLIM cases in the Americas. Demographic, epidemiological, clinical, radiological, and laboratory data were extracted. The main characteristics and results were also compared according to the country's gross national income.

**Results:** A total of 47 articles were included, resulting in 93 cases. The etiological agent was described in 86 (92.5%) patients. Bacteria were the most prevalent etiology (73; 84.8%). Amongst bacterial diagnoses, more frequent cases were *Mycobacterium tuberculosis* (28; 38.4%); *Corynebacterium* spp. (15; 20.5%); non-tuberculous mycobacteria (13; 17.8%). The cases were reported in eight different countries, with the USA being the country with the highest number of cases (35; 37.6%). Patients from high-income countries group presented a shorter diagnostic time when compared to low, low-middle, and upper-middle-income countries. A greater number of radiographic studies with pathological findings were described in high-income countries.

**Conclusion:** Non-lactational infectious mastitis is a complex public health problem with diagnostic and treatment challenges. Hence, multi-professional approach-based additional studies are recommended on its epidemiology, diagnosis, treatment, and control.

## Introduction

Mastitis is a non-malignant inflammatory breast disease, which may be accompanied by an infection, and affects any anatomical structure of the mammary gland (1–3). Infectious etiologies are more frequent in lactating women (lactational or puerperal mastitis) (4, 5). Non-lactational or non-puerperal infectious mastitis (NLIM) can becaused by different infectious agents (6, 7). *Staphylococcus* is the main genus of bacteria associated with non-lactational infectious mastitis (8, 9), and up to 30% may be polymicrobial (associated, for example, with *Enterobacteriaceae, Peptostreptococcus, Propionibacterium*, and *Bacteroides*) (10). *Mycobacterium tuberculosis*, non-tuberculous mycobacteria (NTM), and *Corynebacterium* spp. are considered rare agents associated with NLIM and may be misdiagnosed as idiopathic granulomatous mastitis (IGM) (11–14).

NLIM presents management challenges, a higher number of relapses and complications, such as fistulas, in addition to more significant morbidity and psychological impact in younger patients (15, 16). The incidence and prevalence of NLIM are challenging to estimate since most studies are published on lactational mastitis, and those regarding NLIM have several limitations in their methodology. The highest prevalence of NLIM occurs in women of reproductive age, whether breastfeeding or not (17–19). Males of any age can be affected as well (20–22). Kamal et al. (23) observed NLIM prevalence of 41.6% in women from outpatient clinics and wards.

The understanding of the causes of NLIM is limited. The literature consists mainly of case reports and small case series, and very few of these refer to cases in the Americas. Consequently, there are limited data on epidemiology, diagnostic approach, and treatment as compared to lactational mastitis, even though it is a significant public health problem, especially in low- and middle-income Latin America and the Caribbean. Therefore, we conducted a systematic review of the published literature regarding NLIM cases reported in this geographical region to comprehend the associated factors. Also, we compared characteristics of patients from low, low-middle and upper-middle-income countries (Latin America) and high-income countries [United States of America (USA) and Canada] according to the World Bank classification based on the gross national income (GNI) per capita [31].

## Methods

**Figure 1:**
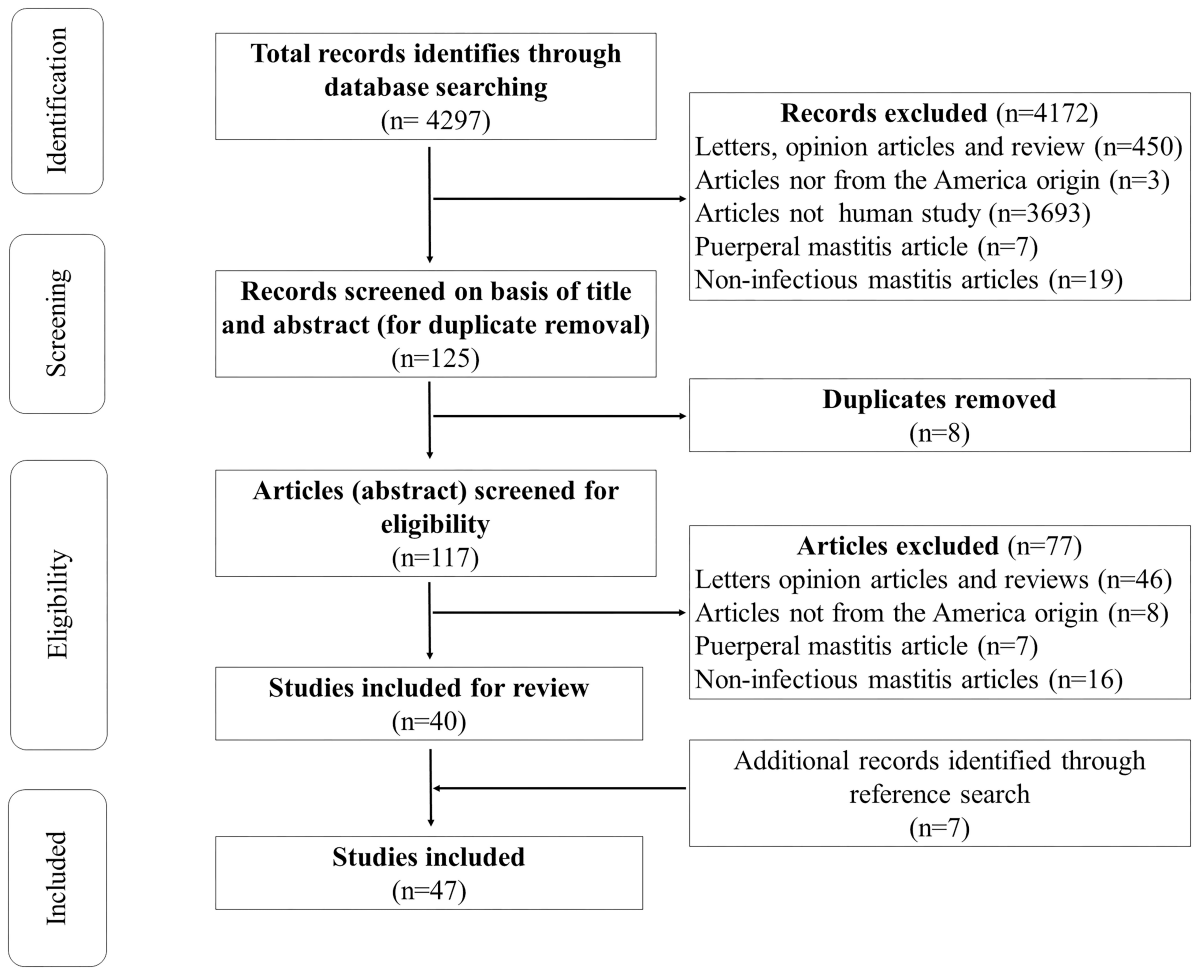
Flowchart of the inclusion of case reports on non-lactational infectious mastitis in studies from the American continent.

The Preferred Reporting Items for Systematic Reviews and Meta-Analyses (PRISMA) guidelines were followed (24). Studies reporting NLIM were systematically selected by two independent reviewers and identified through multiple electronic databases (Medline/PubMed, Lilacs, and Scielo), using the keywords presented in Table 1 as a search strategy. Any disagreements were resolved by consensus. We also assessed the list of references from the included studies to identify other ones that were not initially detected. Figure 1 presents the study selection flow diagram. The last search was performed in October, 2020. No year or language restrictions were applied. Only case reports with primary data were included.

**Table 1:** Search strategy of cases with non-lactational infectious mastitis in the Americas.

**Database**	**Search strategy**
Scielo	Mastitis
Medline/Pubmed Lilacs	Mastitis AND (Americas OR Latin America OR North America OR South America OR Central America OR Antilles OR Anguilla OR Antigua OR Aruba OR Argentina OR Barbuda OR Belize OR Bahamas OR Barbados OR Bolivia OR Bonaire OR Brazil OR Canada OR Caribbean OR Chile OR Colombia OR Costa Rica OR Cuba OR Curacao OR Dominica OR Dominican Republic OR Ecuador OR El Salvador OR Grenada OR Grenadines OR Guadeloupe OR Guatemala OR Guyana OR Haiti OR Honduras OR Jamaica OR Martinique OR Mexico OR Montserrat OR Nevis OR Nicaragua OR Panama OR Paraguay OR Peru OR Puerto Rico OR Saint Kitts OR Saint Lucia OR Saint Vincent OR Suriname OR Surinam OR Trinidad OR Tobago OR United States of America OR USA OR Uruguay OR Venezuela)

Studies were screened initially based on titles and abstracts for data regarding NLIM using pre-defined inclusion and exclusion criteria. Only studies from countries of the Americas were included. Studies were excluded when reporting non-human studies, inconclusive data on mastitis and experimental and basic research approaches. The extracted data from the selected studies included the year of publication, geographical location, data on demography and epidemiology, breast imaging reporting and data system classification (BI-RADS), histopathology, X-rays description, treatment, and relapse rates.

Data were described using descriptive statistics. A Shapiro–Wilk test was used to verify data distribution. Independent *t*-test, Wilcoxon Mann–Whitney and χ^2^ tests were used to compare patients from low, low-middle, and upper-middle-income (Latin American) countries and those from high-income countries (USA and Canada), accordingly. Significance was set as *p* < 0.05. Analyses were performed using STATA® software, version 14 (Stata Corp., College Station, Texas).

## Results

The original search yielded a total of 4,297 potentially eligible studies. After the exclusion of duplicates, screening, and the use of predefined inclusion criteria, a total of 40 studies remained. Seven other studies were added after a reference search of the included studies. A total of 47 (25–71) studies and 93 patients were included (shown in Figure 1); 87 (94.6%) were female, the mean age was 37 (30–52), a history of pregnancy was reported in 24 (26.1%), and 40 (43.5%) presented risk factors (such chronic use of oral contraceptives, long-term steroid use, thoracic surgery, cat scratches, and among others) and/or other associated diseases (shown in Supplementary Table 1). Unilateral localization (78; 83.9%) on the right breast (46; 54.8%) was the most reported. Breast mass (60; 65.2%) and abscess (42; 45.7%) were the most reported clinical findings. One patient was asymptomatic. Table 2 describes other signs and symptoms.

**Table 2:** Clinical findings of cases, etiology, and treatment of non-lactational infectious mastitis in the Americas.

**Variables**	**Total *n* (%)**	**CI 95%**
**Clinical findings**		
***Signs and symptoms previous to hospitalization (n =92)^a^***		
Breast mass	60 (65.2)	0.546–0.749
Abscess	42 (45.7)	0.352–0.564
Fistula	21 (22.8)	0.147–0.328
Breast hardness	11 (13.2)	0.061–0.204
Fever	9 (9.8)	0.046–0.178
Nipple discharge	8 (8.7)	0.038–0.164
Isolated breast pain	4 (4.3)	0.012–0.108
Ulcer	4 (4.3)	0.012–0.108
Breast implant exhibition	3 (3.9)	0.007–0.092
Blisters	1 (1.3)	0.001–0.059
Asymptomatic	1 (1.3)	0.001–0.059
***Breast of occurrence (n=84)***		
Right	46 (54.8)	0.435–0.657
Left	32 (38.1)	0.277–0.493
Bilateral	6 (7.1)	0.027–0.149
***Breast quadrant (n=29)***		
Upper–outer	7 (24.1)	0.103–0.435
Upper–inner	4 (13.8)	0.039–0.317
Lower–outer	3 (10.3)	0.022–0.274
Lower–inner	6 (20.7)	0.080–0.397
More than one quadrant	9 (31.0)	0.153–0.508
**ETIOLOGY (** ***n*** **=** **86)**		
**Bacteria**	**73 (84.8)**	**0.755–0.917**
*Mycobacterium tuberculosis*	28 (38.4)	0.272–0.505
*Corynebacterium* spp.	15 (20.5)	0.120–0.316
Non-tuberculous mycobacteria	13 (17.8)	0.098–0.285
*Mycobacterium fortuitum*	3 (23.1)	0.050–0.538
*Mycobacterium abscessus*	2 (15.4)	0.019–0.454
*Mycobacterium avium*	2 (15.4)	0.019–0.454
*Mycobacterium chelonae*	1 (7.7)	0.002–0.360
*Mycobacterium mucogenicum*	1 (7.7)	0.002–0.360
Not specified	4 (30.8)	0.091–0.614
Other bacteria^b^	17 (23.3)	0.142–0.346
**Fungi**	**9 (10.5)**	**0.049–0.189**
*Histoplasma* spp.	3 (33.3)	0.075–0.700
*Blastomyces* spp.	2 (22.2)	0.028–0.600
*Cryptococcus neoformans*	2 (22.1)	0.028–0.600
*Paracoccidioides brasiliensis*	1 (11.1)	0.002–0.482
*Histoplasma* sp./*Paracoccidioides* sp.	1 (11.1)	0.002–0.482
**Virus:** ***Herpes simplex***	**1 (1.2)**	**0.001–0.063**
**Parasite:*****Sparganum*****sp**.	**1 (1.2)**	**0.001–0.063**
**Normal cutaneous flora**	**1 (1.2)**	**0.001–0.063**
**Not specified**	**1 (1.2)**	**0.001–0.063**
**TREATMENT**		
***Treatment approach (n=90)***		
Only pharmacological therapy	24 (26.7)	0.179–0.125
Pharmacological therapy combined with drainage/surgical procedures	61 (67.8)	0.571–0.772
Only Surgical treatment	5 (5.6)	0.018–0.125
***Pharmacological drugs (n=84)***		
Pharmacological polytherapy	49 (58.3)	0.471–0.690
Pharmacological monotherapy	24 (28.6)	0.192–0.395
Not specified	11 (13.1)	0.067–0.222
***Pharmacological group (n=74)***		
Antituberculous agents	30 (40.5)	0.293–0.526
Fluoroquinolones	9 (12.2)	0.057–0.218
Macrolides	8 (10.8)	0.048–0.202
Sulfonamides	7 (9.5)	0.039–0.185
Tetracyclines	7 (9.5)	0.039–0.185
Antifungal agents	6 (8.1)	0.030–0.168
Beta-lactams	6 (8.1)	0.030–0.168
Cephalosporins	6 (8.1)	0.030–0.168
Lincosamides	5 (6.8)	0.022–0.151
Corticosteroids	4 (5.4)	0.015–0.133
Penicillins	4 (5.4)	0.015–0.133
Immunomodulators	3 (4.1)	0.008–0.114
Aminoglycosides	2 (2.7)	0.003–0.094
Oxazolidinones	2 (2.7)	0.003–0.094
Antiviral agents	1 (1.4)	0.001–0.073
Anti-inflamatories	1 (1.4)	0.001–0.073
Hydroxychloroquine	1 (1.4)	0.001–0.073
Lipopeptides	1 (1.4)	0.001–0.073

a *No signs or symptoms were available in 1 patient;*

b *Staphylococcus sp.: n = 4; Gram-positive bacteria n = 3; Actinomyces sp.: n = 2; Gram-Negative bacteria n = 2: Fusobacterium sp.: n = 2; Finegoldia magna: n = 1; Propionibacterium acne: n = 1; Acinetobacter baumannii: n = 1; Aeromonas hydrophila: n = 1. The bold values mean the main etiology groups*.

The etiological agent was determined in 86 (92.5%) patients. Bacteria were the most frequent etiology (73; 84.8%). *M. tuberculosis* was isolated in 28 (38.4%); 15 (20.5%) by *Corynebacterium spp*.; and 13 cases (17.8%) caused by NTM (shown in Table 2). Fungal (9; 10.5%), viral (1; 1.2%), or parasitic (1; 1.2%) infections were also described. Sixty-six (71.0%) patients underwent invasive procedures (drainage procedures, excisional biopsy, debridement, or resection), all of which combined with pharmacological treatment, except for 5 (5.6%) patients (shown in Table 2). Primarily, epidemiological risk and biopsy results guided treatment. The majority of patients were treated with polypharmacotherapy (shown in Table 2).

Demographics, clinical, diagnostic features, and the causative infectious agent are available for 84 patients (shown in Table 3). Most cases were identified through biopsy alone (41; 48.8%). Different signs and symptoms are related to distinct etiological agents. The median treatment time was 24 weeks (IQR: 4–24). The longest time to elucidate the diagnosis was in cases of tuberculous mastitis (TBM) with a median of 28 weeks (IQR: 16–32; shown in Table 3). Twenty-three cases (27.4%) were of suspected breast cancer; among these, TBM caused 13 (46.4%). Seventy-one (84.5%) cases reported complete remission. Ten (11.9%) relapses were described (shown in Table 3 and Supplementary Table 1). Different imaging methods were used alone or combined. Amongst these patients, 13 (15.5%) individuals had abnormal chest X-rays, and 23 (27.4%) were either classified as BI-RAD IV or BI-RAD V (shown in Table 3). A total of 78 (83.9%) patients underwent biopsy. Granulomas (65/78, 83.3%; 19 were caseous and 46 non-caseous) and necrotic tissue (32/78, 41.0%) were the most prevalent findings. Other biopsy findings are presented in Supplementary Table 1.

**Table 3:** Demographic and clinical characteristics of NLIM patients infected with different pathogens.

**Variables**	***Mycobacterium tuberculosis* (*n* = 28)**	**Non-tuberculous mycobacteria (*n* = 13)**	***Corynebacterium spp*. (*n* = 15)**	**Other bacteria^a^ (*n* = 17)**	**Fungi (*n* = 9)**	**Viruses (*n* = 1)**	**Parasites (*n* = 1)**	**Total of patients^b^ (*n* = 84)**
**Demographics**								
Sex F, *n* (%)	24 (85.7)	13 (100)	15 (100)	15 (88.2)	9 (100)	1 (100)	1 (100)	78 (92.9)
Age (mean ± SD)	48.1 ± 19.7	32.8 ± 10.6	37.6 ± 10.9	34.8 ± 16.3	44.4 ± 16.5	50	64	41.0 ± 16.9
Pregnancy history, *n* (%)	2 (7.1)	3 (23.1)	8 (53.3)	6 (35.3)	N/A^c^	0 (0.0)	N/A	19 (22.6)
Risk factors, *n* (%)	9 (32.1)	10 (83.3)	4 (26.7)	10 (58.8)	3 (33.3)	1 (100)	N/A	37 (44.0)
**Signs and symptoms previous to hospitalization**								
Breast mass, *n* (%)	17 (60.7)	6 (46.2)	11 (73.3)	13 (76.5)	8 (88.9)	1 (100)	1 (100)	57 (67.9)
Abscess, *n* (%)	13 (46.4)	8 (61.5)	6 (40.0)	9 (52.9)	3 (33.3)	0 (0.0)	0 (0.0)	39 (46.4)
Fistula, *n* (%)	12 (42.9)	4 (30.8)	0 (0.0)	2 (11.8)	1 (11.1)	0 (0.0)	0 (0.0)	19 (22.6)
Breast hardness (%)	3 (10.7)	3 (23.1)	1 (6.7)	2 (11.8)	1 (11.1)	0 (0.0)	0 (0.0)	10 (11.9)
Fever, *n* (%)	4 (14.3)	2 (15.4)	1 (6.7)	2 (11.8)	0 (0.0)	0 (0.0)	0 (0.0)	9 (10.7)
Nipple discharge, *n* (%)	2 (7.1)	2 (15.4)	1 (6.7)	1 (5.9)	0 (0.0)	1 (100)	0 (0.0)	7 (8.3)
Ulcer, *n* (%)	1 (3.6)	1 (7.7)	1 (6.7)	0 (0.0)	1 (11.1)	0 (0.0)	0 (0.0)	4 (4.8)
BIE^d^, *n* (%)	0 (0.0)	3 (23.1)	0 (0.0)	0 (0.0)	0 (0.0)	0 (0.0)	0 (0.0)	3 (3.6)
Blisters, *n* (%)	0 (0.0)	0 (0.0)	0 (0.0)	1 (5.9)	0 (0.0)	0 (0.0)	0 (0.0)	1 (1.2)
Isolated breast pain, *n* (%)	0 (0.0)	0 (0.0)	1 (6.7)	0 (0.0)	0 (0.0)	0 (0.0)	0 (0.0)	1 (1.2)
**Diagnosis and treatment**								
Time to close diagnosis in weeks median (IQR)	28 (16–32)	8 (5–12)	8 (2–20)	3 (1–6)	12 (5–22)	4	N/A	12 (4–28)
Treatment time in weeks median (IQR)	24 (24–24)	26 (24–48)	4 (3–10)	3 (2–24)	30 (1–52)	1	N/A	24 (4–24)
Abnormal chest X-ray, *n* (%)	7 (25.0)	1 (7.7)	N/A	1 (5.9)	4 (44.4)	N/A	N/A	13 (15.5)
BIRAD IV or V results on mammography or ultrasound, *n* (%)	16 (57.1)	1 (7.7)	3 (20.0)	2 (11.8)	1 (11.1)	0 (0.0)	0 (0.0)	23 (27.4)
Final diagnosis by biopsy, *n* (%)	18 (64.3)	1 (7.7)	9 (60.0)	5 (29.4)	7 (77.8)	1 (100)^e^	1 (100)	41 (48.8)
Final diagnosis by culture, *n* (%)	4 (14.3)	7 (53.8)	4 (26.7)	9 (52.9)	1 (11.1)	N/A	0 (0.0)	25 (29.8)
Final diagnosis by both biopsy and culture, *n* (%)	4 (14.3)	2 (15.4)	2 (13.3)	3 (17.6)	1 (11.1)	0 (0.0)	0 (0.0)	12 (14.3)
Final diagnosis by molecular methods, *n* (%)	3 (7.1)^f^	2 (7.7)^g^	2 (13.3)^h^	1 (5.9)^i^	0 (0.0)	0 (0.0)	0 (0.0)	8 (9.5)
Final diagnosis by a clinically compatible frame, *n* (%)	0 (0.0)	2 (16.7)	0 (0.0)	0 (0.0)	0 (0.0)	0 (0.0)	0 (0.0)	2 (2.4)
**Clinically or imagological suspicious for malignancy**, ***n*****(%)**	13 (46.4)	0 (0.0)	4 (26.7)	3 (17.6)	3 (33.3)	0 (0.0)	0 (0.0)	23 (27.4)
**Clinical outcomes**								
Surgical resections, *n* (%)	12 (42.9)	11 (84.6)	12 (80.0)	9 (52.9)	7 (77.8)	0 (0.0)	1 (100)	52 (61.9)
Relapse, *n* (%)	0 (0.0)	3 (23.1)	4 (26.7)	3 (17.6)	0 (0.0)	0 (0.0)	0 (0.0)	10 (11.9)
**GNI^j^ per capita classification countries**								
Latin America (LAc^k^)	19 (67.9)	8 (61.5)	3 (20.0)	4 (23.5)	4 (44.4)	0 (0.0)	1 (100)	39 (46.4)
USA/Canada (NAc^l^)	9 (32.1)	5 (38.5)	12 (80.0)	13 (76.5)	5 (55.6)	1 (100)	0 (0.0)	45 (53.6)

a *Other bacteria: Not specified gram-positive bacteria, not specified gram-negative bacteria, Staphylococcus sp., Actinomyces sp., Finegoldia magna, Propionibacterium acnes, Fusobacterium sp., Acinetobacter baumannii, Aeromonas hydrophila;*

b *Completeness of data with just patients with confirmed etiological agent (an etiological agent from two NAc's patients were not specified or described as “normal cutaneous flora,” they were excluded of this table);*

c *N/A, Not data available;*

d *BIE, breast implant exposure;*

e *histological findings accompanied by immunohistochemical studies;*

f *1/3, biopsy, culture, and PCR [polymerase chain reaction] combined;*

g *1/2, culture and PCR combined;*

h *2/2, culture and PCR combined;*

i *1/1, culture and PCR combined;*

j *GNI, gross national income;*

k *LAc, Latin-American countries;*

l *NAc, North American countries*.

**Figure 2:**
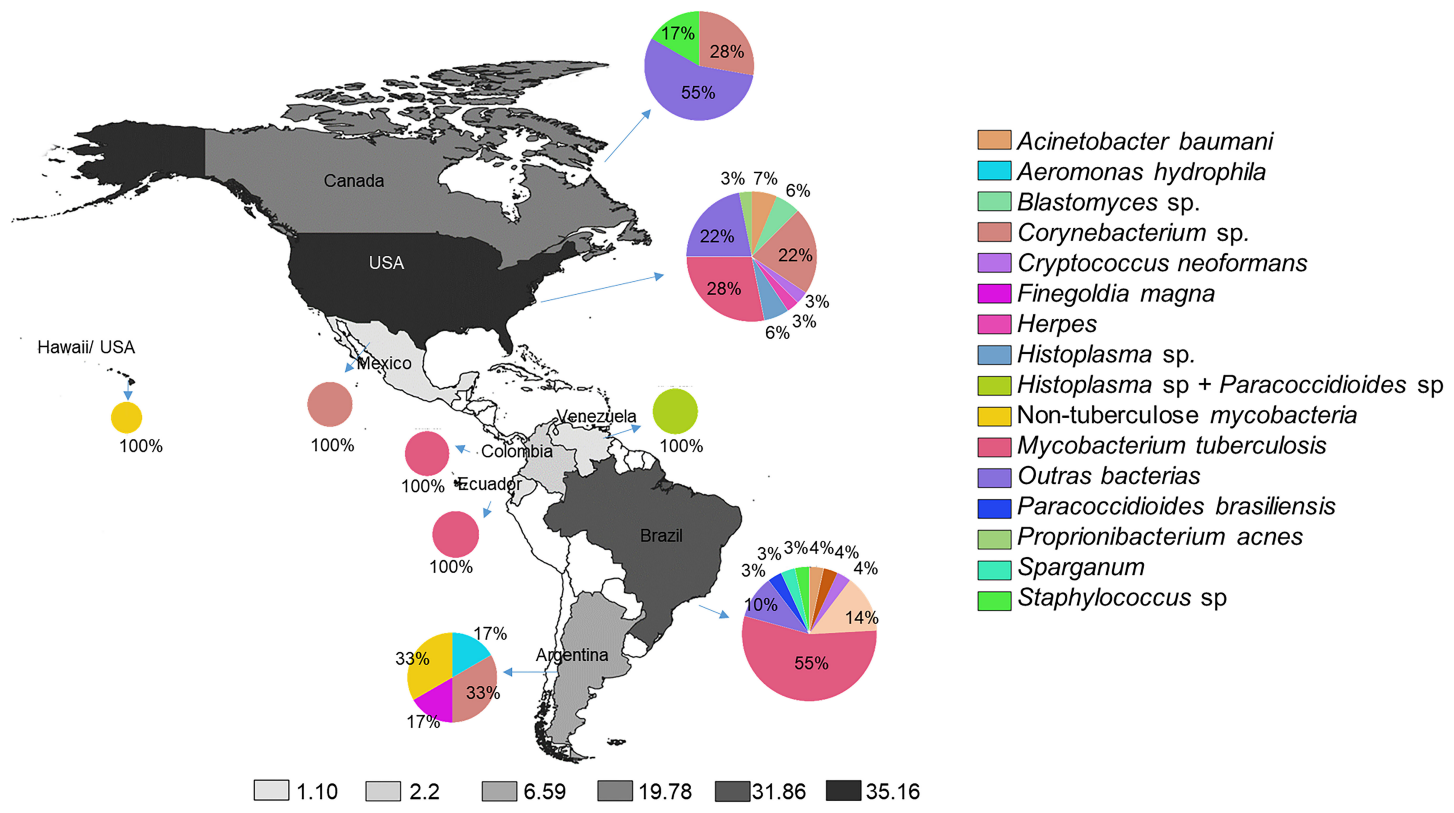
The prevalence of non-lactational infectious mastitis in American countries and the register of etiological infection agents.

The cases were reported in eight different countries, mostly from the USA (35, 37.6%; shown in Figure 2). The Latin-American countries reported a more significant number of TBM and NTM cases. Fungi, *Corynebacterium* spp., and other different bacteria were more frequent in the USA/Canada group (shown in Table 3 and Figure 2). When comparing the two groups of countries categorized by their annual GNI per capita (shown in Table 4), a statistically significant difference was established in terms of the sex most affected by NLIM, the feminine sex (*p* = 0.044). Patients from the high-income countries presented a shorter diagnostic time than low-middle and upper-middle-income countries (*p* = 0.017). Radiographic studies with pathological findings were more described in high-income countries (*p* = 0.004). The diagnosis was confirmed by biopsy in a more significant percentage in Latin American countries (*p* = 0.029).

**Table 4:** Population characteristics, clinical, diagnostic management, and treatment according to high and middle/low-income countries.

**Variables**	**All (*n* = 92)**	**High-income countries group (*n* = 52)**	**Low, low-middle, and upper-middle-income countries (*n* = 40)**	**Completeness *N* (%)**	***p*-value**
**Demographics**					
Sex F, *n* (%)	87 (94.6)	47 (90.4)	40 (100)	92 (100)	**0.044**
Age median(IQR)	37 (30–52)	37 (30–50)	38 (29–54)	91 (98.9)	0.774
Pregnancy history, *n* (%)	24 (26.1)	21 (40.4)	3 (7.5)	36 (39.1)	0.074
Risk factors, *n* (%)	40 (43.5)	25 (48.1)	15 (37.5)	58 (63.0)	0.760
**Diagnosis and treatment**					
Time to close diagnosis in weeks median (IQR)	14 (4–28)	8 (4–20)	24 (8–32)	60 (65.2)	**0.017**
Treatment time in weeks median (IQR)	24 (4–28)	24 (3–36)	24 (24–24)	65 (70.7)	0.188
Abnormal Chest X-ray, *n* (%)	13 (14.1)	9 (17.3)	4 (10.0)	40 (43.5)	**0.004**
Confirmed microorganism, *n* (%)	85 (92.4)	46 (88.5)	39 (97.5)	92 (100)	0.105
Final diagnosis by Biopsy, *n* (%)	42 (45.7)	21 (40.4)	21 (52.5)	74 (80.4)	**0.029**
Final diagnosis by Culture, *n* (%)	25 (27.2)	15 (28.8)	10 (25.0)	54 (58.7)	0.675
**Clinically or imagological suspicious for malignancy**, ***n*****(%)**	24 (26.1)	14 (26.9)	10 (25.0)	88 (95.7)	0.965
**Surgical resections**, ***n*****(%)**	59 (64.1)	38 (73.1)	21 (52.5)	89 (96.7)	**0.028**
**Relapse**, ***n*****(%)**	11 (12.0)	9 (17.3)	2 (5.0)	87 (94.6)	0.057

*The bold values mean that they are statistically significant*.

## Discussion

This study describes the epidemiology, clinical aspects, diagnostics, management, and etiological agents of reported NLIM and compares subjects regarding their country's GNI per capita. Female patients of reproductive age are the most affected by NLIM (17, 72), and only a few reviews have reported cases in men (73, 74). In terms of age and sex distribution, the findings in this study coincide with the prevalence reported (92.9% female) in a study on breast tuberculosis in the Republic of Togo (73).

NLIM is characterized by local inflammatory symptoms and a generally unilateral breast mass, as reported by different studies from Asia and Europe (74–76). Both breast mass and unilateral lesions were found with similar prevalence in this study, although abscess was the most frequent clinical manifestation in Latin American countries. Nair et al. (17) conducted a retrospective study in India in which no case was clinically or radiologically suspected of being malignant, which differs from findings reports in the present study.

The diagnosis of NLIM remains a significant challenge for clinicians in the Americas. The duration of signs and symptoms before a definitive diagnosis may vary significantly. Time to diagnosis was longer in countries of low and middle-GNI per capita in this report compared to those from Asian and European countries (17, 75). A possible reason for this situation could be the lack of clinical suspicion by health-care professionals or the lack of adequate diagnostic techniques (13, 77), especially when comparing the numbers of cases reported in countries of the European continent (78). In addition, culturally related barriers to reproductive health, including breast care, may interfere with the results described in this review (79–81).

Overall, most imaging studies aim to delimitate breast lesions or rule out possible pathologies of malignant origin. Few articles reported the use of imaging techniques for diagnostic purposes on breast infection (82, 83). The number of patients that underwent mammography in this manner (32.3%) was higher than the data described (17.9%) in a Chinese hospital (83). However, the percentage of ultrasound exams was lower than the reported in a Turkish study, in which 100% of the patients underwent ultrasound (82). The use of low-cost imaging techniques, such as ultrasonography, could be an interesting first-line approach for such use in low- and middle-income countries.

The histopathological investigation was the most reliable pathogen identification tool. Granuloma was the most frequent description among patients undergoing a biopsy, which was higher than the one found (21%) in an Indian study on tuberculous mastitis (74). Almost half the cultures in this review reported microorganisms, which was lower than an Irish cohort (88.9%) of cases of NLIM (9). Also, the number of positive cultures in patients from the USA and Canada was higher than in Latin-Americans.

Milk stasis can facilitate the development of lactational mastitis which, together with the gastrointestinal and skin microbiota of the mother and infant, increases the risk of appearance of it (19), however, the most frequent etiological causes of lactational and non-lactational mastitis are led by gram-positive organisms (9). In the Asian continent, *Staphylococcus aureus* was the leading etiologic cause of non-lactating breast infections (8, 84). In this review, Mycobacterium tuberculosis was responsible for the most cases, and although tuberculous mastitis was first described in the 19th century (85) and is considered a rare clinical presentation, it is estimated to occur in up to 4% of patients in endemic countries (86), suggesting the persistence of tuberculosis in the Americas as a public health problem, and since many articles conducted in the Americas focus on tuberculous or granulomatous mastitis (6, 12, 87), it is not surprising that *M. tuberculosis* figures as an important cause of NLIM. Other non-tuberculous mycobacteria may also cause NLIM, as previously reported in India and England (88, 89). Likewise, NLIM cases caused by *Corynebacterium* spp. were reported in Europe (90, 91), both of which were described as the leading causes of mastitis in this review. In 1990, Edmiston et al. (22) also reported other etiological agents that were correlated as other etiological agents of NLIM.

The length of treatment depends on the underlying infectious cause. Several case reports lacked data on the duration of treatment. The treatment time for TM was slightly more than eight months in a Korean study by Seo et al. (13). However, our findings depicted a longer treatment time for this pathology in countries from the Americas. This was similar for *Corynebacterium* spp. infections compared to data from a study conducted in New Zealand (11).

Different management approaches were also found in this study. In a Turkish study, 12 (38%) patients were only treated with surgery, while one (3.2%) was treated with medication (92). In contrast, in our findings, a lower number of cases underwent surgery, and the majority to antimicrobial drugs. Other cases required treatment with a combination of drug therapy and drainage procedures. A more significant amount of patients were treated similarly (9). Overall, patients were prescribed medications from different pharmacological groups, which varied according to the etiological agent and clinical presentation. This variety of drugs has also been reported in Saudi Arabia, where *S. aureus* was the most prevalent etiological agent (84).

Cases of NLIM relapse have been reported in European countries in 11–38.3% of cases (9, 15). The relapse rate obtained in this study was very similar. Most patients in our series reside in high-income countries, and none of them was in the TM group. This may be explained by the fact that recurrences of TM are rare since treatment usually produces a definitive cure (13, 75). Moreover, gram-positive bacteria, such as *Corynebacterium* spp., and gram-negative bacteria were more prevalent in patients from the USA and Canada, with such cases presenting a significant number of relapses (72).

This study has several limitations: infrequent clinical presentations are more likely to be published, leading to publication bias, and undermining real-life prevalence estimation and clinical depiction.; also, assessing disease prevalence or clinical outcomes among several studies with different designs, hypotheses, objectives, methodologies may lead to a lack of data standardization, which is an expected limitation to this type of study; these may also affect case management, which may have further influenced the results of the present study; and finally, the lack of systematic reporting from the included studies hampers comprehensive data analysis and completeness.

## Conclusion

NLIM is a complex disease and presents difficulties in diagnosis and treatment due to various confounding factors as epidemiological, etiological, and clinical aspects. This study summarizes the different epidemiological and clinical aspects of NLIM on the American continent. Tuberculous mastitis, NTM, and cystic neutrophilic granulomatous mastitis (CNGM) due to *Corynebacterium* spp. were the leading infectious causes of NLIM. No publication has described the many facets and features of NLIM in both the Latin American and high-income American countries. Despite a paucity of references discussing NLIM, the results reported in this study demonstrate that it remains a public health problem. The long period from the onset of symptoms to diagnosis shows the importance of a multi-professional approach. Prospective cohorts are necessary to have a greater comprehension of the NLIM, including remote locations such as the Amazon.

## Data Availability Statement

The raw data supporting the conclusions of this article will be made available by the authors, without undue reservation.

## Author Contributions

VC, MC-S, ML, and FV: study concept and design. VC, NC-V, PL, and DB-d-S: acquisition of the data. VC, NV, DB-d-S, and FV: analysis of the data. VC, NC-V, PL, DB-d-S, and FV: drafting of the manuscript. VC, NC-V, PL, DB-d-S, MA, IP, MC-S, ML, and FV: critical revision of the manuscript and approval of final manuscript. All authors contributed to the article and approved the submitted version.

## Conflict of Interest

The authors declare that the research was conducted in the absence of any commercial or financial relationships that could be construed as a potential conflict of interest.

## Publisher's Note

All claims expressed in this article are solely those of the authors and do not necessarily represent those of their affiliated organizations, or those of the publisher, the editors and the reviewers. Any product that may be evaluated in this article, or claim that may be made by its manufacturer, is not guaranteed or endorsed by the publisher.
