# Supplementary material for: Non-lactational Infectious Mastitis in the Americas: A Systematic Review
Source: Front Med (Lausanne). 2021 Aug 2;8:672513. doi: 10.3389/fmed.2021.672513 (PMC8378399; doi:10.3389/fmed.2021.672513)
Supplement: Supplementary file 1 [file Table_1.docx]

**Supplementary Table 1.** Clinical ad demographical characteristics of patients that developed mastitis of infectious etiology from the Americas.

| **Ref.** | **Country** | **Age** | **Pregnancy history** | **Risk factors** | **Time to close diagnosis (Weeks)** | **Signs and symptoms** | **Treatment time (Weeks)** | **Biopsy** | **Etiology** | **Surgical treatment** | **Pharmacological treatment** | **Relapse** |
| --- | --- | --- | --- | --- | --- | --- | --- | --- | --- | --- | --- | --- |
| Al-Qattan, 1990 (1) | Canada | 36 | N/A^1^ | N/A | 13 years | Abscess + Fistula | N/A | Inflammation and cystic degeneration | Chronic mastitis (No microorganism identified ) | Yes | Cloxacillin + topical antibiotics + drainage procedures | No |
| Arango-Fererira, 2018 (2) | Colombia | 9 | No | None | 40 | Breast hardness + Abscess + Fistula + + Ulcer | 32 | Non-caseous granuloma and acute inflammation | *Mycobacterium tuberculosis* (Culture + PCR) | No | RIPE^2^ | No |
| Bhatty, 2016 (3) | USA | 64 | N/A | N/A | 11 | Breast mass + Abscess | N/A | Necrotizing granuloma | *Mycobacterium tuberculosis* (Culture) | No | RIP | No |
| Bianco*.*, 2009 (4) | Brazil | 32 | Yes | Chronic contraceptive use | 12 | Breast mass + Breast hardness | N/A | Granuloma, epithelioid histiocytes and lymphocytes | *Mycobacterium tuberculosis* (Clinical and radiological pattern) | No | RIP | No |
| Brickman, 2005 (5) | Hawaii/ USA | 32 | N/A | Breast Implant | 1 | Breast hardness + Abscess | 24 | N/A | Non-tuberculous Mycobacteria  *Mycobacterium chelonae* (Culture) | Yes | Clarithromycin + TMP/SMX^3 +^ drainage procedures | No |
| Castello, 2007 (6) | Argentina | 32 | N/A | Bilateral reduction mammoplasty | 10 | Abscess | 2 | N/A | *Finegoldia magna* (Culture) | No | Cefadroxil + drainage procedures | No |
| Chambô Filho, 2000 (7) | Brazil | 72 | N/A | None | N/A | Abscess | 144 | Giant cell and fungus yeast | *Paracoccidioides*  *Brasiliensis* (Biopsy) | Yes | TPM/SMX + drainage procedures + Sulfadoxine | No |
| Conde, 2015 (8) | Brazil | 12 | No | None | 1 | Abscess + Breast hardness | 2 | N/A | N/A | No | Cefadroxil + drainage procedures | No |
| Conde, 2015 (8) | Brazil | 12 | No | None | 1 | Abscess + Breast hardness | 2 | N/A | Acute mastitis:  *S. haemolyticu*s (Culture) | No | Cefadroxil + drainage procedures | No |
| Cuervo, 2013 (9) | Colombia | 34 | N/A | None | 16 | Fistula + Fever + Breast hardness | 24 | Granuloma | *Mycobacterium tuberculosis* (PCR+) | No | RIPE | No |
| Cunningham, 2003 (10) | USA | 48 | N/A | HIV/ Previous TB/ HCV/ IV Drug user | 12 | Breast mass + abscess | 48 | Caseous necrotizing granuloma | Non-tuberculous Mycobacteria:  *Mycobacterium avium* complex (Culture) | Yes | Rifabutin + Isoniazid + Ethambutol + Azithromycin | Yes |
| Da Silva, 2005 (11) | Brazil | 73 | Yes | N/A | 32 | Breast mass | 24 | Caseous necrotizing granuloma and Langhans giant cells | *Mycobacterium tuberculosis* (Biopsy) | Yes | RIP | No |
| Da Silva, 2009 (12) | Brazil | 54 | N/A | N/A | 40 | Abscess + Fistula | 24 | Caseous necrotizing granuloma and Langhans giant cells | *Mycobacterium tuberculosis* (Biopsy) | No | RIP | No |
| Da Silva, 2009 (12) | Brazil | 36 | N/A | N/A | 28 | Breast nodule | 24 | Caseous necrotizing granuloma and Langhans giant cells | *Mycobacterium tuberculosis* (Biopsy) | Yes | RIP | No |
| Da Silva, 2009 (12) | Brazil | 73 | N/A | N/A | 20 | Breast nodule | 24 | Caseous necrotizing granuloma and Langhans giant cells | *Mycobacterium tuberculosis* (Clinical and radiological pattern) | Yes | RIP | No |
| Da Silva, 2009 (12) | Brazil | 42 | N/A | N/A | 24 | Abscess + Fistula | 24 | Caseous necrotizing granuloma and Langhans giant cells | *Mycobacterium tuberculosis* (Biopsy) | No | RIP | No |
| Da Silva, 2009 (12) | Brazil | 32 | N/A | N/A | 48 | Abscess + Fistula | 24 | Caseous necrotizing granuloma and Langhans giant cells | *Mycobacterium tuberculosis* (Biopsy) | No | RIP | No |
| Da Silva, 2009 (12) | Brazil | 53 | N/A | N/A | 32 | Abscess + Fistula | 24 | Caseous necrotizing granuloma and Langhans giant cells | *Mycobacterium tuberculosis* (Biopsy) | No | RIP | No |
| Da Silva, 2009 (12) | Brazil | 72 | N/A | N/A | 28 | Breast nodule | 24 | Caseous necrotizing granuloma and Langhans giant cells | *Mycobacterium tuberculosis* (Biopsy) | Yes | RIP | No |
| Da Silva, 2009 (12) | Brazil | 40 | N/A | N/A | 32 | Abscess + Fistula | 24 | Caseous necrotizing granuloma and Langhans giant cells | *Mycobacterium tuberculosis* (Biopsy) | No | RIP | No |
| Da Silva, 2009 (12) | Brazil | 24 | N/A | N/A | 28 | Abscess + Fistula | 24 | Caseous necrotizing granuloma and Langhans giant cells | *Mycobacterium tuberculosis* (Biopsy) | No | RIP | No |
| Da Silva, 2009 (12) | Brazil | 78 | N/A | N/A | 36 | Breast nodule | 24 | Caseous necrotizing granuloma and Langhans giant cells | *Mycobacterium tuberculosis* (Biopsy) | Yes | RIP | No |
| Da Silva, 2009 (12) | Brazil | 25 | N/A | N/A | 40 | Abscess + Fistula | 24 | Caseous necrotizing granuloma and Langhans giant cells | *Mycobacterium tuberculosis* (Biopsy) | No | RIP | No |
| Da Silva, 2009 (12) | Brazil | 23 | N/A | N/A | 24 | Abscess + Fistula | 24 | Caseous necrotizing granuloma and Langhans giant cells | *Mycobacterium tuberculosis* (Biopsy + Ziehl-Neelsen staining) | No | RIP | No |
| Da Silva, 2009 (12) | Brazil | 32 | N/A | N/A | 28 | Abscess + Fistula | 24 | Caseous necrotizing granuloma and Langhans giant cells | *Mycobacterium tuberculosis* (Biopsy) | No | RIP | No |
| Da Silva, 2009 (12) | Brazil | 70 | N/A | Previous TB | 32 | Breast nodule | 24 | Caseous necrotizing granuloma | *Mycobacterium tuberculosis* (Biopsy) | Yes | RIP | No |
| Fernández, 2020 (13) | Argentina | 27 | N/A | N/A | N/A | Abscess | 4 | Granulomatous lobar mastitis | *Corynebacterium amycolatum* (Culture + PCR) | Yes | Ampicillin/ sulbactam + TMP-SMX + drainage procedures | No |
| Fernández, 2020 (13) | Argentina | 62 | N/A | None | N/A | Abscess | 1 | N/A | *Corynebacterium pyruviciproducens* (Culture + PCR) | Yes | Cephalexin | No |
| Fred, 1995 (14) | USA | 37 | N/A | HIV/ Previous TB | 3 | Breast mass + Fever | N/A | Granuloma | *Mycobacterium tuberculosis* (Biopsy + Culture) | No | RIPE | No |
| Gamblin, 2005 (15) | USA | 36 | N/A | Cat scratch | 6 | Breast nodule | 3 | Necrotizing granuloma | Gram negative bacteria (Biopsy: Steiner’s stain) | Yes | Ciprofloxacin | No |
| García-Lozano, 2012 (16) | Argentina | 53 | N/A | Type 1 DM/ Uterine leiomyo- sarcoma | N/A | Blisters + Fever | N/A | Vasculitis + Interstitial inflammatory infiltrate | *Aeromonas hydrophila* (Culture) | N/A | N/A | N/A |
| Goldman, 1995 (17) | USA | 59 | N/A | N/A | 1 | Breast mass | 12 | Macrophages and yeast inclusion inflammation | *Cryptococcus neoformans* (Biopsy: mucicarmine staining + AgCr) | Yes | Fluconazol | No |
| Hale, 1985 (18) | USA | 31 | N/A | Previous TB | 20 | Breast mass + Nipple Discharge + Abscess | 24 | Granuloma | *Mycobacterium tuberculosis* (Biopsy) | Yes | Rifampicin + Isoniazid | No |
| Johnson, 2016 (19) | Mexico | 34 | N/A | N/A | 56 | Breast mass + Abscess | 16 | Granuloma and fat necrosis | *Corynebacterium kroppenstedtii* (Culture) | Yes | Doxycycline + Clindamycin + Amoxicillin/ Clavulanic + Linezolid + Ciprofloxacin + drainage procedures | Yes |
| Kamyab, 2016 (20) | USA | 29 | Yes | None | 8 | Breast mass + Breast hardness + Abscess | N/A | Granuloma, epithelioid histiocytes and Langhans giant cells | Non-tuberculous Mycobacteria:  *M. fortuitum* (Culture) | No | Ciprofloxacin + TMP/SMX + Linezolid + Prednisone + drainage procedures | Yes |
| Krueger, 2019 (21) | USA | 28 | Yes | NA | NA | Breast mass + Ulcer | 32 | Deep dermal infiltrate with epithelioid and multinucleated Langerhans-type giant cells with numerous lymphocytes, plasma cells, focal aggregates of neutrophils and focal caseation | *Mycobacterium mucogenicum*  (culture and PCR) | No | TMP-SMX + clarithromycin | No |
| Lehman, 2017 (22) | USA | 44 | N/A | Type 2 DM | 44 | Breast mass | 24 | Caseous necrotizing granuloma, epithelioid histiocytes and Langhans giant cells | *Mycobacterium tuberculosis* (PCR+) | Yes | RIPE | No |
| Lizaso, 2011 (23) | Argentina | 50 | N/A | Breast implant/ SLE^4^ | N/A | Breast hardness +Abscess + Fistula + Fever + Breast implant exposure | 28 | N/A | Non-tuberculous Mycobacteria:  *M. fortuitum* (Culture) | Yes | Ciprofloxacin + TMP/SMX + Amikacin | No |
| Maung, 2020 (24) | Canada | 50 | Yes | Prolactinoma/  Hypothyroidism | N/A | Breast mass | N/A | Neutrophilic and granulomatous inﬂammation | Gram-positive bacillus: *Corynebacterium* sp*.* (Biopsy, suggestive histologic features) | Yes | Doxycycline | No |
| Maung, 2020 (24) | Canada | 43 | Yes | None | N/A | Breast mass | N/A | Neutrophilic and granulomatous inﬂammation | Gram-positive bacillus: *Corynebacterium* sp*.* (Biopsy, suggestive histologic features) | Yes | Antibiotics | No |
| Maung, 2020 (24) | Canada | 33 | Yes | None | N/A | Breast mass | N/A | Neutrophilic and granulomatous inﬂammation | *Corynebacterium kroppenstedti* (Biopsy: Gram stain and culture) | Yes | Antibiotics | Yes |
| Maung, 2020 (24) | Canada | 35 | No | Polycystic ovarian syndrome/ type II DM/ obesity | N/A | Isolated breast pain | N/A | Neutrophilic and granulomatous inﬂammation | Gram-positive bacillus: *Corynebacterium* sp*.* (Biopsy, suggestive histologic features) | Yes | Antibiotics | No |
| Maung, 2020 (24) | Canada | 54 | Yes | Prolactinoma/ hypothyroidism | N/A | Breast mass | N/A | Neutrophilic and granulomatous inﬂammation | Gram-positive *cocci* (Biopsy: Gram stain) | Yes | Antibiotics | No |
| Maung, 2020 (24) | Canada | 30 | Yes | DM/ smoking | N/A | Breast mass | N/A | Early palisading granulomas | Gram-positive bacillus: *Corynebacterium* sp*.* (Biopsy, suggestive histologic features) | Yes | Antibiotics + drainage procedures | No |
| Maung, 2020 (24) | Canada | 25 | No | Ex-smoker | N/A | Breast mass | N/A | Early palisading granulomas with histiocytes and central neutrophilic infiltrates | No microorganism identified | Yes | Antibiotics | No |
| Maung, 2020 (24) | Canada | 37 | Yes | None | N/A | N/A | N/A | Neutrophilic and granulomatous inﬂammation | Gram-negative *bacilli* (Biopsy: Gram stain) | N/A | N/A | N/A |
| Maung, 2020 (24) | Canada | 43 | Yes | None | N/A | Isolated breast pain | N/A | Neutrophilic and granulomatous inﬂammation | No microorganism identified | Yes | Antibiotics | No |
| Maung, 2020 (24) | Canada | 39 | Yes | Charcot-Marie-Tooth disease with ataxia | N/A | Isolated breast pain | N/A | Neutrophilic and granulomatous inﬂammation | Steiner stain positive (No microorganism specified*)* | Yes | Antibiotics | No |
| Maung, 2020 (24) | Canada | 32 | Yes | None | N/A | Breast mass | N/A | Neutrophilic and granulomatous inﬂammation surrounding clear cystic spaces (lipid droplets) | Coagulase negative *Staphylococcus* (Biopsy: Gram stain and culture) | Yes | Antibiotics + drainage proceudres | Yes |
| Maung, 2020 (24) | Canada | 57 | Yes | N/A | N/A | Breast pain + erythema | N/A | Neutrophilic and granulomatous inﬂammation | No microorganism identified | N/A | N/A | N/A |
| Merino-Alado, 2020 (25) | Venezuela | 51 | N/A | Contact with barnyard birds and farming | 16 | Breast mass + ulcer + fistula + breast hardness | 52 | Non-necrotizing granuloma, histologically formed by epithelioid histiocytes with small intracellular yeast | Histoplasma sp and Paracoccidioides sp. (Biopsy) | No | Itraconazole | No |
| Mohr, 2014 (26) | USA | 15 ds. | N/A | N/A | 1 day | Abscess + Fever + Breast hardness + Fistula | 1 | N/A | Acute mastitis: *Acinetobacter baumani/ haemolyticus* (Culture) | No | Ceftazidime + drainage procedures | No |
| Moreira1997 (27) | Brazil | 64 | N/A | N/A | N/A | Breast mass | N/A | Necrotizing granuloma and eosinophils | *Sparganum* (Biopsy) | Yes | Surgery | No |
| Palmero, 2004 (28) | Argentina | 39 | Yes | DM | 96 | Breast mass + Nipple discharge + Fistula | 60 | N/A | Non-tuberculous Mycobacteria:  *M. fortuitum* (Culture) | No | Kanamycin + Doxycycline + Ciprofloxacin + TMP-SMX | No |
| Payne, 2006 (29) | Brazil | 36 | N/A | HIV/ HBV/ HCV | 20 | Breast mass | N/A | Necrotizing granuloma, epithelioid histiocytes, Langhans giant cells and budding yeast form | *Histoplasma* sp. (Biopsy and Culture) | Yes | Amphotericin B | No |
| Pereira, 2010 (30) | Brazil | 48 | N/A | Breast Implant | 12 | Breast nodule + Nipple discharge + Abscess | 48 | Epithelioid histiocytes, Langhans giant cells and vascular formation. | Non-tuberculous Mycobacteria:  *M. avium* (PCR+) | Yes | Clarithromycin + Ethambutol | Yes |
| Qiao, 2018 (31) | USA | 49 | N/A | Rheumatoid Arthritis + Previous latent TB treatment | 20 | Breast mass + Nipple discharge | 24 | Necrotizing granuloma and epithelioid histiocytes | *Mycobacterium tuberculosis* (Clinical, radiological and histopathological pattern) | No | RIPE | No |
| Ramos-Barbosa, 2004 (32) | Brazil | 46 | N/A | Corticosteroid Therapy | 24 | Breast nodule | 48 | Non-caseating granuloma and fungal spherical encapsulated elements. | *Cryptococcus neoformans* (Biopsy and AgCr) | Yes | Ketoconazole | No |
| Renshaw, 2011 (33) | USA | 54 | N/A | N/A | 8 | Breast mass | 4 | Granuloma and neutrophilic cystic inflammation | Gram-positive bacillus: *Corynebacterium* sp. (Biopsy, suggestive histologic features) | Yes | Tetracycline | No |
| Renshaw, 2011 (33) | USA | 22 | N/A | Nipple piercing | 2 | Breast mass | 2 | Granuloma and Neutrophilic cystic inflammation | Gram-positive bacillus: *Corynebacterium* sp*.* (Biopsy, suggestive histologic features) | Yes | Doxycycline | No |
| Renshaw, 2011 (33) | USA | 27 | N/A | N/A | N/A | Abscess | 4 | Neutrophilic cystic inflammation | Gram-positive bacillus: *Corynebacterium* sp. (Biopsy, suggestive histologic features) | Yes | Tetracycline | No |
| Reyes, 1999 (34) | USA | 68 | N/A | N/A | 4 | Breast mass + Fever | 24 | Mixed cell inflammatory exudate with clusters of epithelioid histiocytes | Empyema necessitatis: *Mycobacterium tuberculosis* (Culture) | No | Rifampicin + Isoniazid | No |
| Reyes, 1999 (34) | USA | 47 | N/A | IV drug user + Smoking | 4 | Breast mass + Abscess | 6 | Acute inflammatory exudate with necrotic debris | Empyema necessitatis: *Actinomyces israeli* and *Actinobacillus actino- mycetemcomitans* (Biopsy and culture) | No | Penicillin  + drainage procedures | No |
| Salfelder et al., 1975 (35) | USA | 30 | N/A | N/A | 8 | Breast mass + Abscess | N/A | Granuloma, epithelioid histiocytes, Langhans giant cells and budding yeast cells | *Blastomyces dermatitidis* (Biopsy) | Yes | Amphotericin B + drainage procedures | No |
| Salfelder et al., 1975 (35) | USA | 21 | N/A | N/A | N/A | Breast nodule | N/A | Necrotizing granuloma, epithelioid histiocytes and Langhans giant cells | *Histoplasma capsulatum* (Biopsy) | Yes | Surgery | No |
| Salfelder et al., 1975 (35) | USA | 55 | N/A | N/A | 24 | Breast mass | N/A | Necrotizing granuloma, epithelioid histiocytes and Langhans giant cells | *Histoplasma capsulatum* (Culture) | Yes | Surgery | No |
| Sánchez-Miño, 2018 (36) | Ecuador | 45 | N/A | None | 2 | Breast mass + Abscess + Fistula | 24 | Non caseating necrotizing granuloma, epithelioid histiocytes and Langhans giant cells | *Mycobacterium tuberculosis* (Biopsy and Culture) | Yes | RIPE | No |
| Shoyele et al., 2018 (37) | USA | 28 | Yes | Nipple piercing | N/A | Breast mass + abscess | 44 | Neutrophilic cystic granuloma | *Proprionibacterium acnes* (Culture) | Yes | Antibiotics + drainage procedures | No |
| Shoyele, 2018 (37) | USA | 36 | Yes | None | N/A | Breast mass | N/A | Neutrophilic cystic granuloma | Gram-positive bacillus: *Corynebacterium* sp. (Biopsy, suggestive histologic features) | No | Topical anti inflammatory | Yes |
| Shoyele, 2018 (37) | USA | 53 | Yes | Previous mastitis | N/A | None | N/A | Neutrophilic cystic granuloma | No microorganism identified | No | Expectant treatment | No |
| Shoyele, 2018 (37) | USA | 41 | Yes | None | N/A | Breast mass + Nipple discharge + Abscess | 36 | Neutrophilic cystic granuloma | *Corynebacterium amycolatum* (Biopsy and culture) | Yes | Cefuroxime + Prednisone + Methotrexate Dalbavancin + Daptomycin + Hydroxy-chloroquine | Yes |
| Shoyele, 2018 (37) | USA | 46 | Yes | None | N/A | Breast mass + Nipple discharge | 40 | Neutrophilic cystic granuloma | Normal cutaneous flora | Yes | Antibiotics + drainage procedures | N/A |
| Shoyele, 2018 (37) | USA | 36 | Yes | None | N/A | Breast mass | 24 | Neutrophilic cystic granuloma | Gram-positive bacillus (Biopsy) | Yes | Antibiotics + Prednisone + Methotrexate | Yes |
| Shoyele, 2018 (37) | USA | 45 | No | None | N3/A | Breast mass | 44 | Neutrophilic cystic granuloma | Gram-positive bacillus (Biopsy) | Yes | Antibiotics + Prednisone + Methotrexate | Yes |
| Silva, 2011 (38) | Brazil | 67 | N/A | Aortic surgery | 1 | Breast mass + Fistula + Abscess | 60 | Necrosis | *Actinomyces europaeus* (Biopsy, culture and PCR) | Yes | Amoxicillin/ Clavulanic + drainage procedures | No |
| Soo, 2000 (39) | USA | 50 | No | Multiple cyst aspirations | 4 | Nipple discharge + Breast cysts | 1 | Neutrophilic and lymphocytic infiltrate | Herpes simplex (Immunohisto-chemical) | No | Aciclovir | No |
| Stary, 2011 (40) | USA | 33 | Yes | None | 2 | Breast mass + Abscess + Fever + Breast hardness | 4 | Granuloma, necrosis and Langhans giant cells | *Corynebacterium* sp*.* (Culture) | Yes | TMP/SMX + Penicillin G and Vancomycin + Doxycycline | N/A |
| Thompson, 1997 (41) | USA | 58 | N/A | Previous TB/ Lung squamous cell carcinoma | 4 | Breast mass + Fever | N/A | Acute inflammatory exudate, necrosis and epithelioid histiocytes | *Mycobacterium tuberculosis* (Biopsy and culture) | Yes | Anti-tuberculous drugs (Not specified) | No |
| Trop et al., 2011 (42) | Canada | 17 | N/A | Smoking | N/A | Breast mass + Abscess | 3 | Chronic inflammation | *Staphylococcus aureus* (Culture) | Yes | Cloxacilin + drainage procedures | No |
| Trop et al., 2011 (42) | Canada | 25 | N/A | N/A | N/A | Abscess + Fistula | 68 | N/A | No microorganism identified | Yes | Cephalexin + Cloxacilin + Clindamycin + drainage procedures | Yes |
| Trop et al., 2011 (42) | Canada | 36 | N/A | Smoking | N/A | Breast mass + Abscess | 2 | N/A | *Bacteroides + Fusobacterium* (Culture) | No | Clindamycin + drainage procedures | No |
| Trop et al., 2011 (42) | Canada | 37 | N/A | N/A | N/A | Breast mass | 3 | Inflammatory changes. | *Staphylococcus aureus* (Culture) | No | Cloxacilin + Clindamycin + drainage procedures | No |
| Trop et al., 2011 (42) | Canada | 22 | Yes | Smoking + nipple piercing | N/A | Breast mass + Abscess | 4 | N/A | *Fusobacterium + Peptostreptococcus* (Culture) | Yes | Clindamycin + drainage procedures | No |
| Trupiano, 2001 (43) | USA | 17 | N/A | Nipple Piercing | 8 | Breast mass | N/A | Non-necrotizing and necrotizing granuloma | Non-tuberculous Mycobacteria:  *M. abscessus* (Culture) | Yes | Surgery | No |
| Vanek et al., 1970 (44) | USA | 30 | N/A | N/A | 8 | Breast mass + Abscess | N/A | Granuloma, epithelioid histiocytes and Langhans giant cells | *Blastomyces* sp. (Biopsy) | Yes | Intrafocal Anphotericin B + Surgery | No |
| Wajnberg, 2011 (45) | Brazil | 23 | N/A | Breast implant | 5 | Abscess + Fistula | N/A | N/A | Non-tuberculous Mycobacteria:  *M. abscessus* (Culture) | Yes | Ciprofloxacin | No |
| Wajnberg, 2011 (45) | Brazil | 31 | N/A | Breast implant | 2 | Fistula | 18 | Necrotizing granuloma, epithelioid histiocytes and Langhans giant cells | Non-tuberculous Mycobacteria (Clinical and histopathological features) | Yes | Ciprofloxacin+ Clarithromycin | No |
| Wajnberg, 2011 (45) | Brazil | 34 | N/A | Breast implant | 4 | Abscess | 12 | N/A | *Mycobacterium* sp. (BAAR +) | Yes | Ciprofloxacin+ Clarithromycin | No |
| Wajnberg, 2011 (45) | Brazil | 23 | N/A | Breast implant | 9 | Abscess + Breast implant exposure | 24 | N/A | No microorganism identified | Yes | Clarithromycin + Ciprofloxacin | No |
| Wajnberg, 2011 (45) | Brazil | 24 | N/A | Breast implant | 24 | Breast implant exposure | 24 | N/A | No microorganism identified | Yes | Clarithromycin + Ciprofloxacin | No |
| Wang, 2020 (46) | USA | 37 | Yes | N/A | 20 | Breast mass + ulcer | N/A | Lobulocentric granulomas with mixed inflammation and cystic spaces lined by neutrophils | Gram-positive bacillus: *Corynebacterium* sp*.* (Biopsy, suggestive histologic features) | No | β-lactam antibiotics | No |
| Wilson et al., 1990 (47) | USA | 83 | No | Chronic osteomyelitis due to *M. tuberculosis* | 120 | Breast mass | 96 | Necrotizing granuloma | *Mycobacterium tuberculosis* (Culture) | No | RI | No |
| Wilson et al., 1990 (47) | USA | 66 | No | Latent TB infection | 3 | Breast mass | 72 | Caseous necrotizing granuloma | *Mycobacterium tuberculosis* (Culture) | Yes | Ciprofloxacin + Ethionamide + Pyrazinamide | No |

^1^N/A: No information available/applicable. ^2^ RIPE: Rifampin, Isoniazid, Pyrazinamide, Ethambutol.  ^3^ TMP/SMX: Trimethoprim/Sulfamethoxazole. ^4^ SLE: Systemic lupus erythematosus.

1. Al-Qattan, M.; M. B.; Robertson, G.A.; Phil M. FRCS. Bilateral Chronic Infection of the Lactosebaceous Glands of Montgomery. Ann Plast Surgey. 1990;25(6):491–3.

2. Arango-Ferreira C, Zapata-Muñoz CM, Gotuzzo E. Images in clinical tropical medicine: cutaneous tuberculosis presenting as mastitis in a prepubertal girl. Am J Trop Med Hyg. 2018;99(6):1360–1.

3. Bhatty O, Waters D, Wilka N, Samuel S, Horne J, Vivekanandan R. A Rare Manifestation of Tuberculosis Presenting in the United States. Case Rep Infect Dis. 2016;2016:1–4.

4. Bianco SR, Gurgel RL, Tavares MDA. Aspectos radiológicos da tuberculose primária da mama: relato de caso e revisão de literatura. Rev Soc Bras Med Trop. 2009;42(2):203–5.

5. Brickman M, Parsa AA, Parsa FD. *Mycobacterium cheloneae* infection after breast augmentation. Aesthetic Plast Surg. 2005;29(2):116–8.

6. Castello, L.; Bou, M.; Bazzana, M. S.; Predari SC. Absceso mamario no puerperal por *Finegoldia magna*. Rev Argent Microbiol. 2007;39:95–8.

7. Chambô Filho A, Borges FLL, Cintra LC, Martins RM. Mastite por Paracoccidioidomicose: Relato de Caso. Rev Bras Ginecol e Obs. 2000;22(9):593–6.

8. Conde DM. Treatment approach for breast abscess in nonlactating adolescents. Int J Gynecol Obstet [Internet]. 2015;128(1):72–3. Available from: http://dx.doi.org/10.1016/j.ijgo.2014.08.009

9. Cuervo, Sonia; Bonilla, Diego; Murcia, Martha; Hernández, Johana; Gómez J. Mastitis tuberculosa. Biomédica. 2013;33:36–41.

10. Cunningham CO, Selwyn PA. Case Report: Mastitis Due to Mycobacterium avium Complex in an HIV-Infected Woman Taking Highly Active Antiretroviral Therapy. AIDS Patient Care STDS. 2003;17(11):547–50.

11. Da Silva BB, Dos Santos LG, Costa PVL, Pires CG, Borges AS. Clinical case report: Primary tuberculosis of the breast mimicking carcinoma. Am J Trop Med Hyg. 2005;73(5):975–6.

12. da Silva BB, Lopes-Costa P V., Pires CG, Pereira-Filho JD, dos Santos AR. Tuberculosis of the breast: analysis of 20 cases and a literature review. Trans R Soc Trop Med Hyg. 2009;103(6):559–63.

13. Fernandez L V., Fortuny AS, Rodriguez EF. Corynebacterium pyruviciproducens and Corynebacterium amycolatum mastitis in immunocompetent no breastfeeding women. Rev Argent Microbiol. 2020;(xx):4–7.

14. Fred HL. An Enlarging Breast Mass in an HIV-Seropositive Woman. Hosp Pract. 1995;30(5):31–2.

15. Gamblin TC, Nobles-James C, Bradley RA, Katner HP, Dale PS. Cat scratch disease presenting as breast mastitis. Can J Surg. 2005;48(3):254–5.

16. García-Lozano T. Lesiones vesículo-ampollosas en un paciente oncológico inmunodeprimido Vesiculobullous lesions in an immunocompromised cancer patient. Rev Argent Microbiol. 2012;31(March):46009.

17. Goldman M, Pottage JC. Cryptococcal infection of the breast. Clin Infect Dis. 1995;21(5):1166–9.

18. Hale JA, Peters GN, Cheek JH. Tuberculosis of the breast: Rare but still extant. Am J Surg. 1985;150(5):620–4.

19. Johnson M, Plongla R, Leone P, Gilligan P. The brief case: Recurrent granulomatous mastitis due to  *Corynebacterium kroppenstedtii* . J Clin Microbiol. 2016;54(8):1938–41.

20. Kamyab A. Granulomatous lobular mastitis secondary to Mycobacterium fortuitum . World J Clin Cases. 2016;4(12):409.

21. Krueger K, Guggina LM. Mycobacterium mucogenicum skin and soft tissue infection of the breast mimicking idiopathic granulomatous mastitis. BMJ Case Rep. 2019;12(12):10–3.

22. Lehman, Trang; Damania, Zubin; Tschetter, Clifford; Lehman N. Case Report. J Fam Pract. 2017;66(1):38–41.

23. Lizaso D, García M, Aguirre A, Esposto A. Infección protésica mamaria por Mycobacterium fostuitum em una paciente con lupus eritematoso sistémico. Rev Chil infectología. 2011;28(5):474–8.

24. Maung MH, Bethune GC, Patriquin G, Barnes PJ. Cystic neutrophilic granulomatous mastitis – a review of 12 consecutive cases. Histopathology. 2020;77(5):781–7.

25. Merino-Alado R, Pineda J, Rasquin JH, Landaeta ME, Mata-Essayag S. Granulomatous mastitis due to coinfection with Histoplasma sp. and Paracoccidioides sp.: A case report. Med Mycol Case Rep. 2020;27(January):52–4.

26. Mohr E, Berhane A, Zora JG, Suchdev P. Acinetobacter baumannii neonatal mastitis: A case report. J Med Case Rep. 2014;8(1):1–3.

27. Moreira MAR, de Freitas R, Gerais BB. Granulomatous mastitis caused by sparganum. Acta Cytol. 1997;41(3):859–62.

28. Palmero DJ, Ambroggi MG, Poggi SE, Muñiz HFJ, Aires B. Mastitis por *Mycobacterium fortuitum* en una paciente HIV negativa. Medicina (B Aires). 2004;64:529–32.

29. Payne S, Kim S, Das K, Mirani N. A 36-Year-Old Woman With a Unilateral Breast Mass. Arch Pathol Lab Med. 2006;130(January):1–2.

30. Pereira LH, Sterodimas A. Autologous fat transplantation and delayed silicone implant insertion in a case of mycobacterium avium breast infection. Aesthetic Plast Surg. 2010;34(1):1–4.

31. Qiao Y, Hayward JH, Balassanian R, Ray KM, Joe BN, Lee AY. Tuberculosis mastitis presenting as bilateral breast masses. Clin Imaging. 2018;52(2017):28–31.

32. Ramos-barbosa S, Guazzelli LS. Criptococose mamária manifesta após corticoterapia Cryptococcal mastitis after corticosteroid therapy. Rev Soc Bras Med Trop. 2004;37:65–6.

33. Renshaw AA, Derhagopian RP, Gould EW. Cystic neutrophilic granulomatous mastitis: An underappreciated pattern strongly associated with gram-positive bacilli. Am J Clin Pathol. 2011;136(3):424–7.

34. Reyes C V., Thompson KS, Jensen J. Fine Needle Aspiration Biopsy of Mastitis Secondary to Empyema Necessitatis. Acta Cytol. 1999;43(5):873–6.

35. Salfelder K. Mycotic “pseudotumors” of the breast. Report of four cases. Arch Surg. 1975;110(6):751–4.

36. Sánchez-Miño JI, Ortíz AM, Orozco L, Venegas B, Yepez F, Escalona-Rabaza M. Tuberculosis de mama: Reporte de un Caso. Rev Peru Med Exp Salud Publica Tuberc. 2018;35(2):333–7.

37. Shoyele O, Vidhun R, Dodge J, Cheng Z, Margules R, Nee P, et al. Cystic neutrophilic granulomatous mastitis: A clinicopathologic study of a distinct entity with supporting evidence of a role for Corynebacterium-targeted therapy. Ann Diagn Pathol. 2018;37:51–6.

38. Silva WA, Pinheiro AM, Jahns B, Bögli-Stuber K, Droz S, Zimmerli S. Breast abscess due to *Actinomyces europaeus*. Infection. 2011;39(3):255–8.

39. Scott Soo, Mary; Ghate S. Herpes Simplex Virus Mastitis. Am J Roentgenol. 2000;174(April):1087–8.

40. Stary CM, Lee YS, Balfour J. Idiopathic granulomatous mastitis associated with corynebacterium sp. Infection. Hawaii Med J. 2011;70(5):99–101.

41. Thompson K, Donzelli J, Jense J, Pachucki C, M A, Reyes C. Breast and cutaneous mycobacteriosis: diagnosis by fine-needle aspiration biopsy. Diagn Cytopathol. 1997;17(1):45–9.

42. Trop I, Dugas A, David J, Khoury ME, Boiuleau J-F, Larouche N, et al. Breast Abscesses: evidence-based algorithms for diagnosis, management, and follow-up. Radiographics. 2011;31(6):1683–99.

43. Trupiano JK, Sebek BA, Goldfarb J, Levy LR, Hall GS, Procop GW. Mastitis Due to Mycobacterium abscessus after Body Piercing. Clin Infect Dis. 2001;33(1):131–4.

44. Vanek J, Schwarz J, Hakim S. North American Blastomycosis: A Study of T e n Cases. Am J Clin Pathol. 1970;54(3):384–400.

45. Wajnberg GB, Basile VVD, Prado LGM, Faria J de, Radwanski HN, Pitanguy I. Mycobacteriosis in patients with breast implants: a case review from the Ivo Pitanguy Institute. Rev Bras Cir Plástica. 2011;26(3):482–7.

46. Wang L, Jorns JM. Cystic neutrophilic granulomatous mastitis: Corynebacterium species-associated infection with distinct histology. Clin Microbiol Infect. 2020;(xxxx).

47. Wilson JP, Chapman SW. Tuberculous mastitis. CHEST J. 1990;98(6):1505–9.
